# Duration, Pattern of Breastfeeding and Postnatal Transmission of HIV: Pooled Analysis of Individual Data from West and South African Cohorts

**DOI:** 10.1371/journal.pone.0007397

**Published:** 2009-10-16

**Authors:** Renaud Becquet, Ruth Bland, Valériane Leroy, Nigel C. Rollins, Didier K. Ekouevi, Anna Coutsoudis, François Dabis, Hoosen M. Coovadia, Roger Salamon, Marie-Louise Newell

**Affiliations:** 1 Africa Centre for Health and Population Studies, University of KwaZulu-Natal, Somkhele, South Africa; 2 INSERM, unité 897, Centre de recherche “Epidémiologie et Biostatistique”, Bordeaux, France; 3 Institut de Santé Publique Epidémiologie Développement (ISPED), Université Victor Segalen Bordeaux 2, Bordeaux, France; 4 Division of Developmental Medicine, University of Glasgow, Glasgow, United Kingdom; 5 Department of Paediatrics and Child Health, University of KwaZulu-Natal, Durban, South Africa; 6 ANRS site in Côte d'Ivoire (PAC-CI), Centre Hospitalier Universitaire de Treichville, Abidjan, Côte d'Ivoire; 7 Centre for HIV/AIDS Networking, University of KwaZulu-Natal, Durban, South Africa; 8 Centre for Paediatric Epidemiology and Biostatistics, Institute of Child Health, University College London, London, United Kingdom; University of Cape Town, South Africa

## Abstract

**Background:**

Both breastfeeding pattern and duration are associated with postnatal HIV acquisition; their relative contribution has not been reliably quantified.

**Methodology and Principal Findings:**

Pooled data from 2 cohorts: in urban West Africa where breastfeeding cessation at 4 months was recommended but exclusive breastfeeding was rare (Ditrame Plus, DP); in rural South Africa where high rates of exclusive breastfeeding were achieved, but with longer duration (Vertical Transmission Study, VTS). 18-months HIV postnatal transmission (PT) was estimated by Kaplan-Meier in infants who were HIV negative, and assumed uninfected, at age >1 month. Censoring with (to assess impact of mode of breastfeeding) and without (to assess effect of breastfeeding duration) breastfeeding cessation considered as a competing event. Of 1195 breastfed infants, not HIV-infected perinatally, 38% DP and 83% VTS children were still breastfed at age 6 months. By age 3 months, 66% of VTS children were exclusively breastfed since birth and 55% of DP infants predominantly breastfed (breastmilk+water-based drinks). 18-month PT risk (95%CI) in VTS was double that in DP: 9% (7–11) and 5% (3–8), respectively (p = 0.03). However, once duration of breastfeeding was allowed for in a competing risk analysis assuming that all children would have been breastfed for 18-month, the estimated PT risk was 16% (8–28) in DP and 14% (10–18) in VTS (p = 0.32). 18-months PT risk was 3.9% (2.3–6.5) among infants breastfed for less than 6 months, and 8.7% (6.8–11.0) among children breastfed for more than 6 months; crude hazard ratio (HR): 2.1 (1.2–3.7), p = 0.02; adjusted HR 1.8 (0.9–3.4), p = 0.06. In individual analyses of PT rates for specific breastfeeding durations, risks among children exclusively breastfed were very similar to those in children predominantly breastfed for the same period. Children exposed to solid foods during the first 2 months of life were 2.9 (1.1–8.0) times more likely to be infected postnatally than children never exposed to solids this early (adjusted competing risk analysis, p = 0.04).

**Conclusions:**

Breastfeeding duration is a major determinant of postnatal HIV transmission. The PT risk did not differ between exclusively and predominantly breastfed children; the negative effect of mixed breastfeeding with solids on PT were confirmed.

## Introduction

HIV can be transmitted from mother to infant during pregnancy, delivery or postnatally through breastfeeding, and is a major cause of child mortality in sub-Saharan Africa [1]. Mother-to-child transmission of HIV occurring around delivery can be largely prevented by peri-partum antiretroviral regimens [2]. As a consequence, HIV transmission through breastmilk has emerged as a more important mode of paediatric acquisition in African breastfeeding populations [3], and its prevention remains challenging [4].

Exclusive breastfeeding has been reported to carry a lower postnatal HIV transmission risk than breastfeeding while concurrently feeding other milks, non-milk fluids, and solid foods [5]–[7]. HIV is transmitted throughout the breastfeeding period, and longer duration of breast-feeding is associated with a greater cumulative risk of postnatal HIV transmission [3], [8], [9]. Both breastfeeding pattern and duration are therefore associated with acquisition of HIV, but to date a more precise estimate of postnatal transmission at times points according to breastfeeding exclusivity has not been reliably quantified in the same study. This information is needed to ensure correct and appropriate messages are provided during infant feeding counselling.

To investigate this question, we used a pooled dataset from two studies, designed with a similar study approach, but with two crucial differences with regard to infant feeding practices: an urban West African setting where breastfeeding cessation at 4 months was recommended and where most women did not practise exclusive breastfeeding [10]; and a rural South African setting where emphasis was placed on the promotion of safer breastfeeding practices, resulting in high rates of exclusive breastfeeding, but where breastfeeding duration was much longer [5].

## Methods

### Ethics statement

The Ditrame Plus study was granted ethics permission in Cote d'Ivoire from the ethics committee of the National AIDS Control Programme, and in France from the institutional review board of the ANRS (Agence Nationale de Recherche sur le Sida et les hepatites virales). The Vertical Transmission Study was approved by the Biomedical Research Ethics Committee of the University of KwaZulu-Natal, South Africa. All women included in the Ditrame Plus study and in the Vertical Transmission Study provided written informed consent.

### Study design and interventions to prevent mother-to-child transmission of HIV

We pooled together the data from two cohort studies, of which inclusion procedures and research design have been described in detail elsewhere: the Ditrame Plus study in Côte d'Ivoire [11], [12] and the Vertical Transmission Study (VTS) in South Africa [5], [13].

This pooled analysis was planned from the inception of both studies, which contributed to avoid large differences between studies, with regard to data collection and analysis [14]. This approach allowed for standardized study design and standardized definitions of exposure (see the subsequent “Collection of data on infant feeding practice” paragraph) and confounder variables in both individual studies.

From March 2001 to July 2003, pregnant, HIV-infected, women aged ≥18 years from selected community-run health facilities were enrolled in the Ditrame Plus study in Abidjan, Côte d'Ivoire [11], [12]. All enrolled women received peri-partum antiretroviral zidovudine with or without lamivudine, and single-dose nevirapine to prevent mother-to-child transmission of HIV. Single-dose nevirapine was offered to all infants. Antenatally, two feeding options were discussed: complete avoidance of breastfeeding or exclusive breastfeeding with early cessation from the fourth month. Maternal infant feeding choice was supported, and replacement feeding from birth, or from breastfeeding cessation until 9 months of age, was provided free of charge. Mother-infant pairs were offered clinical, nutritional and psychosocial follow-up in study clinics from birth to two years.

From August 2001 to August 2004, pregnant, HIV-infected, woman aged ≥16 years within selected clinics in rural, semi-urban and urban KwaZulu-Natal, South Africa were enrolled in the VTS [5], [13]. Single-dose nevirapine was offered to all women and infants. Women were counselled antenatally about infant feeding options in line with WHO guidelines [15]. Emphasis was placed on the promotion of exclusive breastfeeding for the first six months for women choosing breastfeeding. From 2002, a six months' supply of infant formula was offered free through the KwaZulu-Natal prevention of mother-to-child transmission programme; women could choose to access this supply from birth or any time in the first 12 months after delivery. Breastfeeding mothers were visited at home by counsellors who supported them in their infant feeding choice every two weeks until the infant was aged 6 months. Clinical follow-up of infants from birth to two years took place in study clinics.

### Collection of data on infant feeding practices

Similar questionnaires were used to collect infant feeding practices in both studies [10], [16], [17]. As recommended [18]–[20], infant feeding practices were recorded as 24-hour and seven-day recall histories detailing all feeds, liquids and non-human milks given to infants for every day of the preceding week. In both studies, this information was recorded by trained field workers who were not involved in infant feeding counselling.

Infant feeding practices were recorded weekly for the first 9 months, and then quarterly until age 24 months in the VTS (41 visits); and weekly until 6 weeks of age, monthly until 9 months of age, and every 3 months until the child's second birthday in the Ditrame Plus Study (18 visits). For this analysis, infant feeding practices in the VTS were obtained only from visits shared with the Ditrame Plus schedule, taking the VTS visit closest to the age at which a Ditrame Plus monthly visit would have been made. We assessed the appropriateness of this in a sensitivity analysis for the VTS only which showed the same feeding behaviour despite the reduced number of visits used (data not shown).

World Health Organization infant feeding definitions were used [21]–[23]. A child who was breastfed at a given age while having never received any other drink, food or non-human milk was exclusively breastfed. Breastfed children having been given water or water-based drinks only were predominantly breastfed from the date of introduction of these fluids. A child who was breastfed at a given age while also having received food-based fluids, solid foods or non-human milk was considered mixed fed from the date of introduction of these fluids or foods. In this paper we distinguish two types of mixed feeding: breastmilk and food-based fluids or solids; and breastmilk and non-human milk.

### Diagnosis of paediatric HIV infection

In the Ditrame Plus Study, venous blood samples were collected within 72 hours of delivery, at 4 to 6 weeks of age, at 2, 3, 6 and 9 months of age, and then every 3 months until 18 months of age. An additional sample was obtained 2 months after breastfeeding cessation. HIV status was established by quantitative HIV RNA assay (Versant bDNA HIV RNA kit version 3.0, Bayer diagnostics, Emeryville, USA, and TaqMan HIV-1 RNA real-time PCR from 2003) [24], [25].

In the VTS, dried blood spot samples were obtained on filter paper within 72 hours of delivery, at 4 to 6 weeks of age, monthly until 9 months of age, and then every 3 months until 18 months of age. HIV status was established by quantitative HIV RNA assay (Nuclisens HIV-1 QT, Organon Teknika, Boxtel, Netherlands, and Nuclisens EasyQ HIV-1, Biomerieux, Boxtel, Netherlands) [26].

In both studies, paediatric HIV infection was defined as a positive plasma HIV-1 RNA PCR at any age. Children with a negative RNA PCR from a sample obtained at age ≥30 days who later became infected were considered to be HIV-infected postnatally [27], [28].

### Statistical methods

This analysis was conducted among live born children of HIV-infected mothers. We excluded infants not tested for HIV infection, with unknown timing of infection or infected during the peri-partum period, and with unknown or imprecise infant feeding practices (at least one recall history missing in the first 2 weeks of life) or formula-fed from birth.

The Turnbull's extension of the Kaplan-Meier procedure to interval-censored data is recommended to assess the postnatal risk of HIV transmission when the length of intervals between HIV tests is long. However a standard Kaplan-Meier approach to the analysis has been shown to be sufficient if most intervals are short (less than 3 months) [28], which was the case in our two studies (data not shown). We thus assessed cumulative HIV postnatal transmission in the first 18 months of life by Kaplan-Meier analysis; association with maternal and infant variables was quantified in a Cox regression analysis [29], [30]. Breastfeeding pattern and duration were considered simultaneously in Cox regression analyses. All multivariable analyses included a dummy variable for study (VTS vs. Ditrame Plus) to account for differing baseline risks between the two cohorts.

Postnatal HIV transmission (beyond 4 weeks of age) was assessed in infants with a negative RNA PCR from a sample obtained at age ≥30 days. The time of acquisition of infection was assumed to be mid-way between the date of the last negative and the first positive HIV RNA test [27].

Survival analysis was conducted to compare HIV postnatal transmission between the two studies. We used two different approaches for censoring to investigate separately the effects of breastfeeding pattern and duration on the postnatal risk of HIV transmission. First, infants were censored on the last day they had been seen in study clinics or on the day they died; this approach allowed us to investigate the impact of breastfeeding duration on the postnatal risk of HIV transmission (as censoring did not depend on breastfeeding duration). Second, infants were censored on the date of breastfeeding cessation, unless they were still being breastfed at the end of the study or if the date of the last negative test was before the date of the breastfeeding cessation plus 30 days, in the latter two cases the infants were censored on the date of the last available negative HIV test. In this second approach, breastfeeding cessation was considered a competing event which allowed the comparison of the two cohorts with regard to the probability of acquisition of HIV postnatally at a given age, which assumes that all children of both studies had been breastfed until that age. This approach allowed us to study the impact of the breastfeeding pattern on the risk of HIV transmission, once the breastfeeding duration had been fully taken into account.

Finally, we calculated smooth estimates of the hazard function taking into account the instantaneous age-specific incidence of postnatal transmission, using the penalized likelihood approach and proportional hazard regression model for interval-censored and left-truncated data (PHMPL) [31]. This analysis considered the competing risk from breastfeeding cessation, and the estimates obtained were expressed per 100 child-years of breastfeeding. Upper and lower confidence bands of the estimate of age-specific incidence were estimated using a Bayesian technique [31].

Statistical analyses were carried out using SAS software (version 9.1; SAS Institute, http://www.sas.com) and the PHMPL computer program developed by the INSERM Research Centre in Epidemiology and Biostatistics of the Bordeaux University (version 1.2; http://www.isped.u-bordeaux2.fr/recherche/biostats/Telechargement/PHMPL/us-Biostats-PHMPL.htm).

## Results

### Cohort profile and baseline characteristics

As shown in Table 1, 2190 HIV-infected women delivered in the VTS (n = 1460) and Ditrame Plus (n = 730) studies, and 1195 breastfed infants who were HIV-negative at or after 30 days were included in the present analysis.

**Table 1:** Cohort profile.

		Vertical Transmission Study (South Africa)	Ditrame Plus (Côte d'Ivoire)
**HIV-infected pregnant women included** 1		**1,775**	**808**
	*exclusion of women with HIV-1 status non confirmed or infected with HIV-2 only*	*9*	*34*
	*exclusion of HIV-1 infected women lost to follow-up or dead before delivery or who had spontaneous abortion*	*306*	*44*
**HIV-1 infected women having delivered**		**1,460**	**730**
**Birth outcomes**		**1,496**	**763**
	*exclusion of second/third born of twins/triplets*	*36*	*33*
	*exclusion of stillbirths*	*60*	*19*
**Single or first live born infants from HIV-infected mother**		**1,400**	**711**
	*exclusion of infants not tested for HIV infection* 2	*193*	*21*
	*exclusion of infants with unknown timing of infection*	*50*	*3*
	*exclusion of infants infected in peri-partum*	*125*	*42*
	*exclusion of infants with imprecise mode of feeding* 3	*50*	*23*
	*exclusion of formula fed infants*	*111*	*298*
**Mother-infant pairs included in the analysis**		**871**	**324**

Vertical Transmission Study, South Africa, 2001–2007 - Ditrame Plus, Côte d'Ivoire, 2001–2005.

1 Vertical Transmission Study: from August 2001 to August 2004, Ditrame Plus: from March 2001 to July 2003.

2 These children died or were lost-to-follow-up before we had an opportunity to test them for HIV infection.

3 At least one recall history missing in the first 2 weeks of life.

Women from the VTS had a higher level of education, were less likely to be employed in the formal economic sector, were less likely to have piped water inside the home, had fewer previous pregnancies and were less likely to be immuno-suppressed than women from Ditrame Plus (Table 2).

**Table 2:** Baseline characteristics of mother-infant pairs.

Characteristics	Subcategory	Vertical Transmission Study (South Africa)	Ditrame Plus (Côte d'Ivoire)	p-value
		N = 871	N = 324	
**Maternal age, median** (IQR)		25 (21–29)	26 (23–30)	0.10
**Maternal education,** n (%)				
	none	60 (7)	141 (43)	<0.001
	primary school	316 (36)	112 (35)	
	secondary school or higher	495 (57)	71 (22)	
**Mother employed in formal economic sector,** n (%)				
	yes	115 (13)	172 (53)	<0.001
	no	750 (87)	152 (47)	
	*missing data*	*6*	*0*	
**Water access,** n (%)				
	piped water inside home	66 (8)	84 (26)	<0.001
	piped water outside home	528 (61)	240 (74)	
	other sources of water	271 (31)	0	
	*missing data*	*6*	*0*	
**Number of previous pregnancies,** median (IQR)		1 (0–2)	3 (2–5)	<0.001
**Peri-partum antiretroviral prophylaxis,** n (%)				
	sdNVP	871 (100)	0	-
	scAZT+sdNVP	0	150 (46)	
	scAZT+sc3TC+sdNVP	0	174 (54)	
**Antenatal maternal CD4 count x10^6^ cells/ml,** median (IQR)		479 (334–647)	400 (270–535)	0.02
	*missing data*	*42*	*2*	
**Antenatal maternal log_10_ plasma HIV RNA viral load,** median (IQR)		3.9 (3.3–4.5)	4.3 (3.7–4.7)	0.23
	*missing data*	*52*	*9*	
**Female child,** n (%)				
	yes	435 (50)	159 (49)	0.78
	no	436 (50)	165 (51)	
**Multiple pregnancy** (twins or triplets), n (%)				
	yes	22 (3)	12 (4)	0.27
	no	849 (97)	312 (96)	
**Low birth weight** (<2,500 g), n (%)				
	yes	84 (10)	87 (27)	<0.001
	no	751 (90)	237 (73)	
	*missing data*	*36*	*0*	

IQR: inter-quartile range; sdNVP: Nevirapine single dose; scZDV: short-course Zidovudine; sc3TC: short-course Lamivudine.

Pearson χ^2^ test or the Fisher exact test were used to compare categorical variables, and the Mann-Whitney U test was used to compare continuous variables.

### Breastfeeding duration and pattern

**Figure 1:**
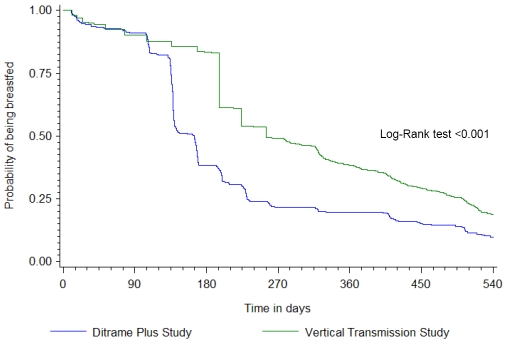
Probability of being breastfed from birth until 18 months of age ^*^. * See Supporting information file S1 for the probability with 95%CI.

Figure 1 shows that 90% of children in both studies were still being breastfed by 3 months of age; but overall breastfeeding duration was shorter in Ivorian than South African children: median durations in months (inter-quartile range) were 4 (3–7) and 7 (5–13) in Ditrame Plus and in the VTS, respectively.

Most infants were exclusively breastfed for at least 3 months in the Vertical Transmission Study and more than one third of them for 6 months, whereas this practice was uncommon in the Ditrame Plus study (Table 3). Although in both studies most infants were exclusively or predominantly breastfed before 3 months of age, thereafter infants in Ditrame Plus were more likely to be mixed fed than infants in the VTS. This practice was associated with breastfeeding cessation and the introduction of complementary feeds which was recommended to occur earlier in Ditrame Plus than in the Vertical Transmission Study. Mixed feeding outside the period of transition when cessation of breastfeeding was occurring with the intentional introduction of complementary feeds (referred to as the weaning process in this paper), was rare in both studies. For instance, 22% of infants had been exposed to any type of mixed feeding by 3 months of age: 3% had been fed with breastmilk plus solids and were not in the process of ‘being weaned’, while the remaining 19% had received breastmilk and non-human milk as part of their weaning process. Similarly, 78% of infants who were mixed fed prior to 6 months completely stopped breastfeeding within 2 weeks of first consuming the solid food or animal milk that transfered them into the mixed feeding category.

**Table 3:** Probabilities^1^ of being exclusively, predominantly breastfed or mixed fed, and not being breastfed from birth until 9 months of age.

From birth until	Probability of being exclusively breastfed (95% CI)		Probability of being exclusively or predominantly breastfed (95% CI)		Probability of being mixed fed (95% CI)		Probability of not being breastfed any more (95% CI)	
	*Vertical Transmission Study*	*Ditrame Plus*	*Vertical Transmission Study*	*Ditrame Plus*	*Vertical Transmission Study*	*Ditrame Plus*	*Vertical Transmission Study*	*Ditrame Plus*
Age 1 Month	0.78	0.23	0.78	0.74	0.22	0.26	0.05	0.06
	(0.75–0.80)	(0.19–0.28)	(0.75–0.80)	(0.68–0.78)	(0.20–0.25)	(0.22–0.32)	(0.04–0.07)	(0.04–0.09)
Age 3 Months	0.66	0.15	0.67	0.55	0.33	0.45	0.10	0.09
	(0.63–0.69)	(0.11–0.19)	(0.64–0.70)	(0.49–0.60)	(0.30–0.36)	(0.40–0.51)	(0.08–0.12)	(0.06–0.13)
Age 6 Months	0.38	0.01	0.39	0.09	0.61	0.91	0.17	0.62
	(0.35–0.42)	(0.001–0.02)	(0.36–0.42)	(0.06–0.13)	(0.58–0.64)	(0.87–0.94)	(0.14–0.19)	(0.56–0.67)
Age 9 Months	0.02	0	0.02	0	0.98	100	0.51	0.79
	(0.01–0.03)		(0.01–0.04)		(0.96–0.99)		(0.48–0.55)	(0.74–0.83)

1 Probabilities were assessed with Kaplan-Meier analysis.

### 18-month postnatal transmission of HIV

**Figure 2:**
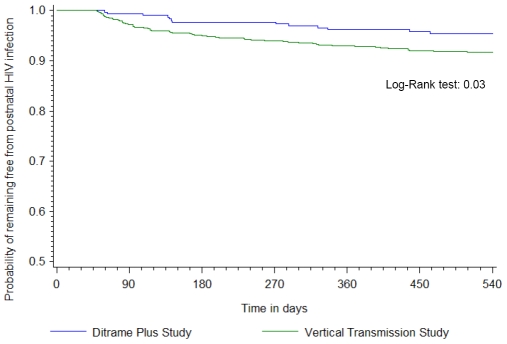
Probability of remaining free from postnatal HIV infection, weaning being not considered as a competing event ^*^. * See Supporting information file S2 for the probability with 95%CI.

**Figure 3:**
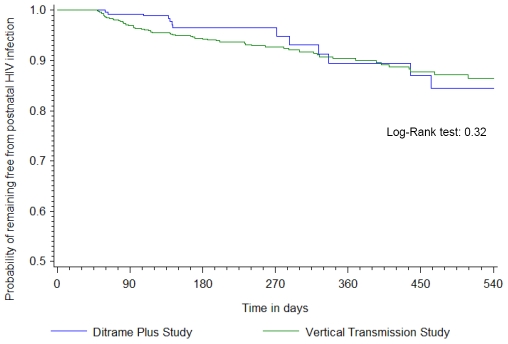
Probability of remaining free from postnatal HIV infection, weaning being considered as a competing event ^* §^. * See Supporting information file S3 for the probability with 95%CI. ^§^ The probability of HIV acquisition postnatally at a given age was calculated assuming that all children had been breastfed until that age.

Overall, 76 cases of confirmed HIV postnatal transmission were diagnosed by 18 months of age, 13 in Ditrame Plus and 63 in the Vertical Transmission Study. The overall 18-month probability of postnatal acquisition of HIV in the Vertical Transmission Study was double that in Ditrame Plus (Figure 2, Table 4). However, the risk was similar in the two studies once duration of breastfeeding had been taken into account in a competing risk analysis (Figure 3). The estimated overall risk of postnatal acquisition of HIV was 9.0/100 child-years of breastfeeding (95% confidence interval [CI], 6.2–11.7).

**Table 4:** Factors associated with postnatal HIV transmission (excluding infant feeding practices), Cox Regression analysis.

Variables	Subcategory	N	Postnatal HIV transmission		Univariable analysis			Multivariable analysis		
			18-month cumulative risk	95% CI	Hazard ratio	95% CI	p	Adjusted hazard ratio	95% CI	p
**Study**										
	Ditrame Plus	324	4.6	2.6–7.8	1	-	0.03	1	-	0.38
	Vertical Transmission Study	871	8.5	6.5–11.5	2.0	1.1–3.6		1.3	0.7–2.5	
**Secondary school or higher**										
	no	629	5.9	4.2–8.3	1	-	0.06	1	-	0.34
	yes	566	8.8	6.7–11.7	1.6	1.0–2.4		1.3	0.8–2.0	
**Mother employed**										
	yes	287	2.8	1.4–5.9	1	-	0.004	1	-	0.01
	no	902	8.8	7.0–11.0	3.1	1.4–6.9		2.9	1.2–6.5	
**Maternal CD4 count**										
	≥200 cells/ml	1032	6.2	4.9–8.0	1	-	0.002	1	-	<0.001
	<200 cells/ml	119	14.4	9.4–21.7	3.2	1.8–5.7		2.9	1.7–5.1	
	missing	44	12.1	4.7–31.5	0.7	0.2–1.9		0.5	0.2–1.5	

### Infant feeding determinants of HIV postnatal transmission

Every baseline characteristic in Table 2 was tested univariably in a Cox regression analysis; variables not detailing infant feeding practices used for adjustment in the following multivariable analyses were chosen on the basis of their statistical significance univariably and are reported in Table 4.

We ran several analyses to investigate the effect of the breastfeeding duration and pattern on the risk of postnatal transmission.

First, we investigated the effect of the breastfeeding duration. The overall risk of postnatal HIV infection was 3.9% (95%CI, 2.3–6.5) among children breastfed for less than 6 months, and 8.7% (95%CI, 6.8–11.0) among children breastfed for 6 months or more (unadjusted hazard ratio: 2.1, 95%CI 1.2–3.7, p = 0.02). Adjustment on study, education level, mother employment status and antenatal maternal CD4 count reduced the hazard ratio to 1.8 (95%CI 0.9–3.4, p = 0.06). Each additional month of breastfeeding beyond 6 months of age was associated with a 1% risk of acquisition of HIV (95%CI, 0.5–1.7).

Second, we compared the postnatal risk of HIV transmission in exclusively and predominantly breastfed children. In individual analyses of postnatal transmission rates for specific durations of breastfeeding (1, 2, 3, 4, 5, or 6 months), the risks among children exclusively breastfed (reference) were very similar to those observed in children predominantly breastfed for the same duration, with adjusted hazard ratios ranging from 0.75 (95%CI, 0.35–1.65) to 1.45 (95%CI, 0.26–8.27) and p-values ranging from 0.35 to 0.98.

Third, we assessed the effect of two different mixed feeding practices on the postnatal risk of HIV transmission. Paradoxically, children simultaneously exposed to breastmilk and non-human milk were less likely to be HIV-infected postnatally than children who were breastfed and unexposed to non-human milk, but exposure to this type of mixed feeding was strongly correlated with shorter breastfeeding cessation, which explained the direction of the association. For instance, the median duration of breastfeeding was shorter by 2 months among children exposed to mixed feeding in the first 3 months of age compared to children exclusively or predominantly breastfed during the same period (6.5 vs. 8.5 months, p<0.001). But, although rare, mixed feeding following the early introduction of solid foods always increased the postnatal risk of acquisition of HIV. Thus, children exposed at least once to solids during the first 2 months of life were 2.9 times more likely to be infected postnatally than children never exposed to solids during the same period, in a competing risk analysis adjusting on the variables in Table 4 (95%CI, 1.1–8.0, p = 0.04). After adjustment on these same variables, very similar results were obtained in an analysis not considering weaning as a competing risk, but taking into account breastfeeding duration as an additional adjustment variable (less vs. more than 6 months): adjusted hazard ratio, 3.6 (95%CI, 1.3–10.0, p = 0.01). The adverse effect of the introduction of solids in the first 2 months of life on the postnatal risk of HIV infection was even stronger among infants of women with baseline CD4 count above 200 cells/ml: adjusted hazard ratio, 4.4 (95%CI, 1.6–12.3, p = 0.005).

Taking into account the instantaneous age-specific incidence of postnatal transmission by infant feeding practices, the estimated postnatal risk of transmission would be 9.0/100 child-years of exclusive breastfeeding (95% support interval [SI], 6.0–12.1), 8.5/100 child-years of predominant breastfeeding (95%SI, 1.2–18.1), and 41.2/100 child-years of breastfeeding plus solids (95%SI, 1.1–74.5).

## Discussion

In this pooled analysis of West and South African cohorts, we compared HIV postnatal transmission rates in two different situations. In South Africa, more women practised exclusive breastfeeding, but breastfeeding duration was longer; duration was shorter in Côte d'Ivoire, but most infants were predominantly breastfed. We show that although crude HIV postnatal transmission rates were twice as high in the South African study than in the Ivorian one, transmission rates were very similar in the two studies once the breastfeeding duration had been taken into account. Our interpretation is that breastfeeding duration is the major determinant of postnatal HIV transmission, and this risk is not substantially different between exclusively and predominantly breastfed children. In addition, with regard to breastfeeding pattern, we confirm the considerable negative effects of mixed breastfeeding with solids.

This pooled study has strengths, but also some limitations that need to be addressed. The two studies constituted a large sample size and were conducted in contexts representative of urban and rural African settings. Moreover, this pooled analysis offered a unique variation of both breastfeeding pattern and duration. Most importantly, the methodology chosen to record infant feeding practices was similar in both studies and more detailed than in previous studies on this subject [6], [7], [32], [33]. For instance, detailed feeding information was collected at 6 weeks, 3 and 6 months only in the Zvitambo trial [7], while we used here feeding information collected 18 times during the first two years of life (with weekly, monthly and then quarterly visits). This strategy minimized the maternal recall bias that could have impaired the estimation of breastfeeding characteristics, both in terms of pattern and duration [18]. The two studies had different short-course peri-partum antiretroviral regimens with the VTS using single-dose nevirapine only and the Ditrame Plus study using zidovudine+/-lamivudine with single-dose nevirapine. It is therefore possible that the more efficacious regimens used in the Ditrame Plus study would have led to reduced transmission in the early postnatal period. The timing of HIV infection was estimated with the same level of precision in both studies, with short intervals between study visits when HIV testing of children was performed. However, the timing of HIV infection was imprecise for 50 children in the VTS and 3 in Ditrame Plus. Most (80%) of these children had a first positive test around 2 months of age with no previous negative test. It is therefore likely that their infection occurred during the peri-partum period, and we did not consider these infants as postnatally infected. Assuming that these children had been infected in-utero or through delivery, the peri-partum transmission rate was 12.5% in the VTS, which is consistent with previous estimates among women exposed to single dose nevirapine during labour [34], [35]. In multivariable analyses, results were adjusted on all baseline characteristic variables which differed between the two studies, and a dummy variable for study was used to account for additional differing baseline risks between the two cohorts. However, we acknowledge that there may have been further confounding factors we could not take into account.

The first key message of this study is the confirmation that mixed feeding, particularly giving breastmilk and solids to infants, is hazardous in terms of postnatal HIV transmission. It is essential that whilst exclusive breastfeeding is promoted, the particular dangers of mixed feeding with solids are emphasised to mothers. We found that the concomitant use of breastmilk and non-human milk was associated with a reduced risk of postnatal HIV transmission. However, in both studies, this infant feeding practice was part of the weaning process as recommended by study protocols, and was therefore immediately preceding breastfeeding cessation. A previous study showed that children simultaneously exposed to breastmilk and non-human milk outside the weaning process (e.g. not immediately before breastfeeding cessation) were more likely to be infected than those exclusively breastfed [36]. These results underline the inherent problem in simultaneously addressing both breastfeeding pattern and duration, as these factors are confounded. Our findings also suggest that the introduction of water-based fluids or fruit juices may not be detrimental in terms of HIV transmission. This finding is consistent with a study conducted in Zimbabwe suggesting that HIV transmission through exclusive and predominant breastfeeding was not different at 18 months (6.94, 95%CI: 2.03–12.89 and 8.56, 95%CI: 5.47–11.63, respectively), although there was a trend towards greater postnatal risk of transmission among predominantly breastfed children [7]. Further support is provided by two other studies showing no difference in mortality between predominantly and exclusively breastfed children [37], [38]. There is no denying that women should be encouraged to give breastmilk only for other reasons linked to maternal and infant health [39]–[41]. However, given that exclusive breastfeeding is rarely practised in African communities [10], [42], [43], unless extensive counselling has been provided [5], [44], [45], it is helpful for policy makers and health workers to appreciate the difference in risks for HIV transmission between predominant feeding with water-based fluids compared to mixed feeding with solids. More specifically, counselling should focus on emphasizing the risks of mixed feeding with solids which are more hazardous than predominant feeding with water-based fluids. Moreover, future studies on HIV and infant feeding should differentiate between predominant and mixed feeding as they appear to have different implications.

This study also shows that breastfeeding duration is a major determinant of transmission, but the public health implications of this finding are highly dependent on the local context. Breastfeeding cessation at 6 months should only occur with appropriate and sustained nutritional counselling; practical support before stopping breastfeeding; access to affordable and nutritionally adequate complementary foods to replace breastmilk after breastfeeding cessation; and careful clinical follow-up of the growth and health of the non-breastfed child [46]–[52]. Therefore, the benefit of breastfeeding cessation in terms of reduction of postnatal HIV transmission has to be balanced with the potential risks for infant health [53].

The risk of HIV transmission through breastmilk is strongly associated with advanced immune deficiency. In our study, this risk was 3.2 (95%CI, 1.8–5.7) and 3.0 (95%CI, 1.9–4.8) times higher among women with respectively baseline CD4 count below 200 and below 350, when compared to women above these thresholds. Although further investigations are needed to explore the balance of benefits and risks related to highly active antiretroviral therapy (HAART) in breastfeeding mothers, it is expected to reduce breastfeeding-associated HIV transmission among women eligible for treatment because of their own health [54], [55]. However, the adverse effect of breastfeeding pattern and duration remains among breastfeeding women who are not eligible for HAART. In our study, among women with CD4 count above 200 cells per ml (a common cut-off for treatment eligibility), breastfeeding beyond 6 months of age tended to be associated with a 1.7 increase (95%CI, 0.9–3.1) of postnatal transmission, while exposure to solids in the first 2 months was associated with a 4.4 increase (95%CI, 1.6–12.3). It is therefore important to continue to promote public health messages on the benefits of optimal infant feeding practices despite the increasing roll-out of HAART among African women.

Despite counselling for breastfeeding cessation at 6 months of age, over 30% of women in the Vertical Transmission Study were still giving some breastmilk at 12 months. There is, therefore, an urgent need to find innovative strategies to retain the benefits of breastfeeding beyond 6 months whilst minimising the HIV transmission risk. Possibilities include: inactivation of the virus with heat treatment [56], [57] or with microbicides [58], use of maternal highly active therapy to lower viral load in breastmilk [59], or even vaccines [60]. Coupled with this, further research is required to support the local production of sustainable complementary foods [61]. Several interventions are presently being investigated in a number of randomised controlled studies and will assist finding solutions to the problem of HIV transmission through breastmilk in Africa. For instance, the administration of antiretroviral drugs to breastfed infants as a post-exposure prophylaxis is an interesting strategy to reduce postnatal HIV transmission [62], [63], especially for children born to women who present late in pregnancy; but this intervention would need to be maintained throughout the breastfeeding exposure and should ideally involve drugs that are not likely to be associated with the development of viral resistance mutations compromising future treatment options for HIV-infected children.

## Supporting Information

Supporting Information File S1 (0.35 MB DOC)

Supporting Information File S2 (0.35 MB DOC)

Supporting Information File S3 (0.34 MB DOC)
