# Supplementary material for: Duration, Pattern of Breastfeeding and Postnatal Transmission of HIV: Pooled Analysis of Individual Data from West and South African Cohorts
Source: PLoS One. 2009 Oct 16;4(10):e7397. doi: 10.1371/journal.pone.0007397 (PMC2759081; doi:10.1371/journal.pone.0007397)
Supplement: Supporting Information File S1 — (0.35 MB DOC) [file pone.0007397.s001.doc]

**Supporting information file S1. Probability (95%CI) of being breastfed from birth until 18 months of age.**

| Probability of being breastfed (95%CI) | Age 3 Months | Age 6 Months | Age 9 Months | Age 12 Months | Age 18 Months |
| --- | --- | --- | --- | --- | --- |
| Ditrame Plus Study | 0.91 | 0.38 | 0.21 | 0.20 | 0.10 |
|  | (0.87-0.94) | (0.33-0.44) | (0.17-0.26) | (0.15-0.24) | (0.07-0.14) |
| Vertical Transmission Study | 0.90 | 0.83 | 0.49 | 0.38 | 0.18 |
|  | (0.88-0.92) | (0.81-0.86) | (0.45-0.52) | (0.35-0.42) | (0.16-0.21) |
