# Supplementary material for: Duration, Pattern of Breastfeeding and Postnatal Transmission of HIV: Pooled Analysis of Individual Data from West and South African Cohorts
Source: PLoS One. 2009 Oct 16;4(10):e7397. doi: 10.1371/journal.pone.0007397 (PMC2759081; doi:10.1371/journal.pone.0007397)
Supplement: Supporting Information File S2 — (0.35 MB DOC) [file pone.0007397.s002.doc]

**Supporting information file S2. Probability (95%CI) of remaining free from postnatal HIV infection, weaning being not considered as a competing event.**

| Probability of remaining free from postnatal HIV infection (95%CI) | Age 3 Months | Age 6 Months | Age 9 Months | Age 12 Months | Age 18 Months |
| --- | --- | --- | --- | --- | --- |
| Ditrame Plus Study | 0.99 | 0.98 | 0.97 | 0.96 | 0.95 |
|  | (0.97-1.00) | (0.96-0.99) | (0.95-0.99) | (0.93-0.98) | (0.92-0.97) |
| Vertical Transmission Study | 0.97 | 0.95 | 0.94 | 0.93 | 0.91 |
|  | (0.96-0.99) | (0.93-0.96) | (0.92-0.95) | (0.91-0.95) | (0.89-0.93) |
